# Equine Herpesvirus-1 Induced Respiratory Disease in Dezhou Donkey Foals: Case Study from China, 2024

**DOI:** 10.3390/vetsci12010056

**Published:** 2025-01-14

**Authors:** Lian Ruan, Liangliang Li, Rongze Yang, Anrong You, Muhammad Zahoor Khan, Yue Yu, Li Chen, Yubao Li, Guiqin Liu, Changfa Wang, Tongtong Wang

**Affiliations:** 1 Liaocheng Research Institute of Donkey High-Efficiency Breeding and Ecological Feeding, Liaocheng University, Liaocheng 252000, China; 2 College of Veterinary Medicine, Shanxi Agricultural University, Taigu 030801, China; 3 College of Veterinary Medicine, Qingdao Agricultural University, Qingdao 266109, China

**Keywords:** respiratory illness, donkey, EHV-1, phylogenetic analyses, pathogenicity

## Abstract

Equine herpesvirus-1 (EHV-1) is a significant pathogen that causes substantial economic losses in the equine industry worldwide, which leads to severe respiratory diseases and abortions in horses. However, reports of EHV-1 infection in donkeys are limited, particularly in China. This case study reported an EHV-1-induced respiratory disease in Dezhou donkey foals in Shandong Province, China, in July 2024. Three one-month-old foals exhibited high fever, nasal discharge, and respiratory distress, with a 100% mortality rate. The causative agent, strain LC126, was isolated from a one-month-old donkey foal exhibiting severe respiratory disease. Phylogenetic analysis of the EHV-1 isolate LC126 showed close similarity to EHV-1. Overall, our study revealed that EHV-1 can cause respiratory distress as well as death in donkeys. The study underscores the emerging threat of EHV-1 in donkeys and highlights the need for veterinarians and breeders to give proper attention to the potential threat of EHV-1 outbreaks.

## Simple Summary

Equine herpesvirus-1 (EHV-1) is known to cause severe respiratory diseases and abortions in horses. However, there is limited information regarding EHV-1 infection in donkeys, particularly in China. The present study reports the isolation of the virus from a one-month-old donkey foal (LC126) exhibiting severe respiratory disease. Genetic analysis revealed that the isolate is closely related to EHV-1. Notably, LC126 was also capable of inducing respiratory distress in a mouse model. These findings provide valuable insights into the pathogenicity of EHV-1 in donkeys.

## 1. Introduction

Equid herpesviruses (EHVs) are a group of significant viral pathogens that pose a persistent threat to the equine industries worldwide [1,2]. To date, nine distinct equid herpesviruses (EHV-1–9) have been identified. Among these, EHV-1–5 primarily infects horses, while EHV-6–8, also known as asinine herpesvirus types 1–3 (AHV-1–3), predominantly affects donkeys [3,4]. EHV-1, first isolated in the United States in 1954, continues to be a widespread pathogen in equine populations globally [5]. EHV-1 is primarily associated with respiratory disorders, abortion, and viral encephalitis in horses [6].

In recent years, the growth of large-scale donkey farming in several regions in China has been accompanied by an increase in respiratory disease and abortion, both of which significantly impact the economic viability of the donkey industry. Interestingly, EHVs have been identified as key contributors to these health issues [7,8,9]. For instance, a study reported a strong correlation between EHV-1 and donkey abortions in Northern Xinjiang, China [10]. Furthermore, Wang et al. documented the association of EHV-8 with respiratory disease and miscarriages in donkeys [11,12]. Notably, reports of EHV-1-induced respiratory disease in donkeys have been rare. In this study, we present a comprehensive report of an EHV-1 infection in donkeys in Shandong Province, China, in 2024.

## 2. Materials and Methods

### 2.1. Herd History and Case Presentation

In July 2024, a respiratory disease outbreak occurred in a large-scale donkey farm located in Shandong Province, China. The affected animals were donkey foals, with three individuals presenting with severe clinical signs, including high fever (41 °C), nasal discharge, respiratory distress (dyspnea), depression, and anorexia. The duration of the illness was approximately five days, and the mortality rate was 100% among the affected foals. A one-month-old dead foal was submitted to the veterinary hospital of Liaocheng University for pathogen identification and diagnostic evaluation.

### 2.2. Nucleic Extraction, PCR Amplification and Sequencing

Nasal swabs and homogenized lung tissue samples from dead foals were collected and resuspended in 2 mL of phosphate-buffered saline (PBS). The samples underwent three freeze–thaw cycles. Subsequently, 1 mL of the PBS suspension was used for viral DNA extraction using the MiniBEST Viral RNA/DNA Extraction Kit (Takara Bio, Beijing, China). Polymerase Chain Reaction (PCR) was employed to detect EHV-1, EHV-4, and EHV-8 in the clinical samples. Specific primers (Table 1) were used for amplification, and PCR products were analyzed by 1% agarose gel electrophoresis. Additionally, another 1 mL of PBS was utilized to extract total RNA using SparkZol Reagent (Sparkjade, Jinan, China). The RNA was reversed into complementary DNA (cDNA) using Prime-Script™ Reverse Transcriptase (Takara Bio, Beijing, China). Furthermore, PCR was performed to detect influenza A virus H3N8 and equine arteritis virus (EAV) in the clinical samples with specific primers (Table 1), and the resulting PCR products were analyzed using the same method described above. Positive amplification products were subsequently sequenced by Sangon Biotech (Shanghai, China) for further analysis.

### 2.3. Pathological Examination

Histopathological analysis was conducted as previously described [13]. In brief, lung tissues were collected from the dead donkey foals and fixed in 10% formalin solution. Following fixation, the tissues were dehydrated, embedded in paraffin, and sectioned into 4 mm thickness. The sections were then stained with hematoxylin and eosin (H&E) for general morphological evaluation. Immunohistochemistry (IHC) was performed to detect EHV-1 infection, utilizing a mouse anti-EHV-1 primary antibody, which was prepared in our laboratory. Finally, tissue samples were examined under a light microscope (Leica DMi8, Wetzlar, Germany), and representative images were captured for analysis.

### 2.4. Isolation of the Virus from Positive Lung Samples

The Rabbit kidney cells (RK-13) were seeded into 6-well plates and incubated overnight. Lung tissues were homogenized in a 5 mL centrifuge tube, followed by the addition of 3 mL of phosphate-buffered saline (PBS) containing penicillin and streptomycin. The tissue suspension was subjected to freeze–thaw cycles three times and subsequently filtered through a 0.22 μm sterilizing filter (Millipore, Merck, Darmstadt, Germany) after centrifugation. The resulting supernatant was then inoculated onto the RK-13 cell monolayer and incubated for 2 h. After the incubation period, the culture medium was replaced with minimum essential medium (MEM) supplemented with 3% fetal bovine serum (FBS) and incubated at 37 °C in a 5% CO_2_ for three days. Cytopathic effects (CPEs) induced by the virus were monitored daily. The supernatants from the third passage were collected and analyzed for the presence of EHV-1 using PCR with specific primers (Table 1). The newly isolated EHV-1 strain was named LC126.

### 2.5. Morphological Observations Using Transmission Electron Microscope

The morphology of EHV-1 was observed as previously described [11]. In brief, the RK-13 cells were seeded into a T75 cm^2^ cell culture flask overnight and infected with LC126 at 1 multiplicity of infection (MOI). These cells were fixed with TEM fixative at 48 hpi and collected by cell scraper and washed with PBS. This was followed by pre-embedding by 1% agarose and post-fixation in 1% osmium tetroxide in 0.1 M PB (pH 7.4) for 2 h at room temperature. Subsequently, dehydration in ethanol (30%, 50%, 70%, 80%, 95%, and 100%), the areas containing cells were block mounted, and the sections were cut into 60–80 nm sections using Leica ultramicrotome (Wetzlar, Germany). Finally, the sections were observed and imaged under a Hitachi transmission electron microscope (Tokyo, Japan).

### 2.6. Indirect Immunofluorescence

Immunofluorescence assays (IFA) were performed as described previously [14,15]. The coverslips were pre-seeded into 12-well cell plates overnight then infected with 0.1 MOI LC126. All cells were fixed at 48 hpi using 75% cold ethanol and sealed with 1% bull serum albumin (BSA), then these cells were incubated with primary antibody mouse EHV-1 convalescent serum and secondary antibodies with DyLight 488-conjugated affiniPure goat anti-mouse IgG + IgM (H+L) (Jackson, PA, USA). Finally, these cells were stained with 4,6-diamidino-2-phenylindole (DAPI) (Sparkjade, Jinan, China), and observed and captured by fluorescence microscope (Leica DMi8, Wetzlar, Germany).

### 2.7. The ORF33 of LC126 Amplification and Phylogenetic Analysis

The *ORF33* sequence from the LC126 isolate was amplified in this study; the reference sequences were downloaded from the GenBank database (http://www.ncbi.nlm.nih.gov/Genbank, accessed on January 2013). The phylogenetic tree was constructed using MEGA 6.0 software (Masatoshi Nei lab, State College, PA, USA) by the neighbor-joining method with 1000 bootstrap replications.

### 2.8. Infection of BALB/c

Ten female, specific pathogen-free BALB/c mice (6 weeks old) were obtained from Pengyue Laboratory Animal Breeding Co., Ltd. (Jinan, China) and randomly divided into infected group and mock group (*n* = 5). BALB/c were inoculated intranasally with LC126 (1 × 10^5^ PFU/mice) or DMEM. Mice of each group were separated into different rooms to prevent cross-infection, and their body weights and clinical features were recorded. All mice were euthanized at 7 dpi via cervical dislocation, and the lung tissues were collected for pathologic analysis examination. Lung tissues of each group were fixed in 10% buffered formalin and embedded in paraffin. Paraffin-embedded tissue sections were used for H&E staining. In addition, the EHV-1 antigen in the lung tissue of different groups of mice was detected with anti-EHV-1 positive serum using IHC.

### 2.9. Statistical Analysis

Statistical analysis was performed using GraphPad Prism version 8.0 (San Diego, CA, USA). Differences between groups were compared using an unpaired Student’s *t*-test. *p* < 0.05 was considered to indicate a statistically significant difference.

## 3. Results

### 3.1. Case Presentation and Pathogen Identification

**Figure 1:**
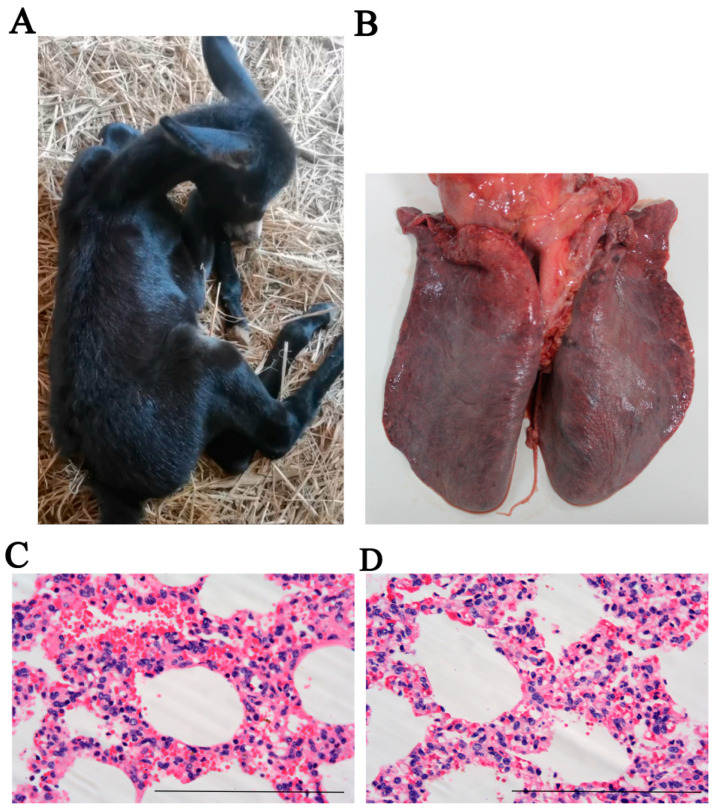
Gross lesions of a respiratory disease killed the foal: (**A**) A respiratory disease in foal donkey; (**B**) Gross change in lungs; (**C**) Hyperemia and hemorrhage in lung; (**D**) Severe interstitial pneumonia.

In July 2024, three one-month-old donkey foals from a large-scale farm in Liaocheng, China, were observed to exhibit severe respiratory distress and subsequently died. The clinical signs, including anorexia, high fever, nasal discharge, dyspnea, and depression, were also observed in infected foals (Figure 1A). Necropsy of one deceased foal revealed a blue/purple discoloration of the lung tissue, with no significant gross abnormalities noted (Figure 1B). Histopathological examination of the lung showed hyperemia and hemorrhage of the alveolar septa (Figure 1C), as well as thickening of the alveolar septa and a notable increase in inflammatory cell infiltration (Figure 1D).

**Figure 2:**
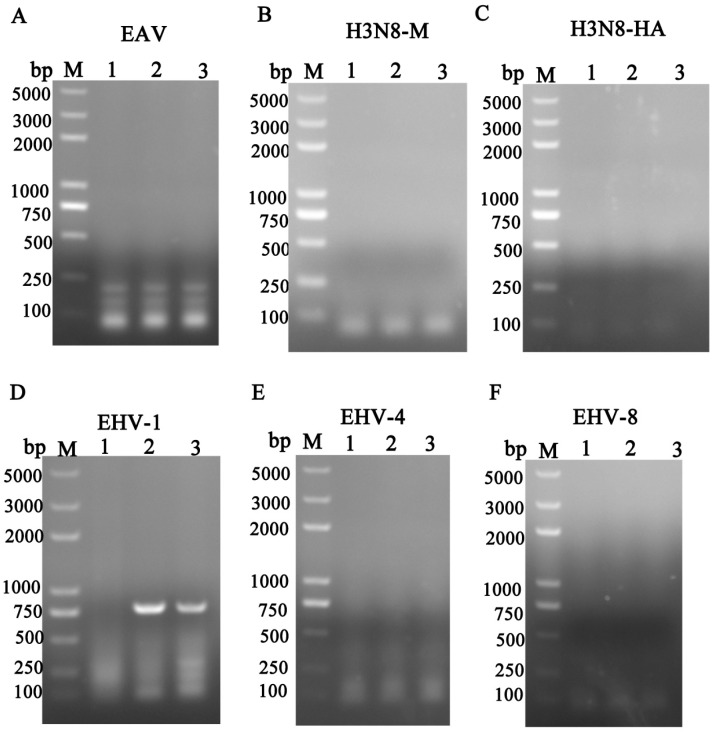
Screening of virus pathogens. Viral DNA/RNA was extracted from different samples: (**A**) EAV; (**B**) H3N8 M; (**C**) H3N8 HA; (**D**) EHV-1; (**E**) EHV-4; (**F**) EHV-8. They were detected by RT/PCR and PCR. Lane M represents a 5000 bp DNA molecular weight ladder. Moreover, 1 represents negative control, 2 represents nose swabs, and 3 represents lung of donkey foal.

To identify the causative pathogen, lung tissue samples were processed and tested for various infectious agents. As shown in Figure 2, PCR testing for EAV (333 bp), H3N8 (595 bp or 224 bp), EHV-4 (1591 bp), and EHV-8 (316 bp) yielded negative results. However, the lung tissue tested positive for EHV-1, with a specific amplification of 792 bp corresponding to the EHV-1 *ORF70* gene.

**Figure 3:**
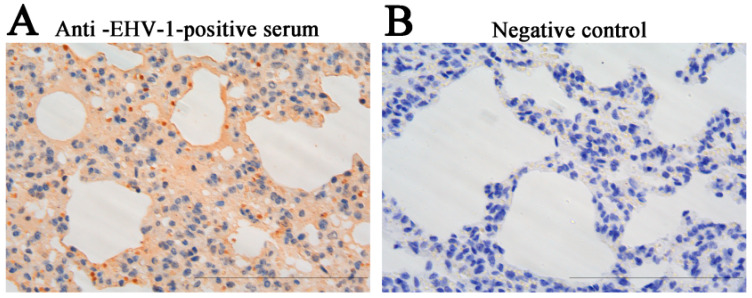
The immunohistochemistry (IHC) detection for EHV-1 in the lung of the donkey. The IHC was performed to detect the EHV-1 antigen in the lungs. The experimental group was treated with mouse anti-EHV-1 positive serum on the lung (**A**). The normal mouse serum-treated group served as a negative control on lung (**B**). Scale bars, 50 μm.

Furthermore, the IHC staining was performed on lung tissue sections of donkey foal to further confirm EHV-1 infection. Positive staining for EHV-1 antigen was observed in the lung tissue, with brown-colored signals indicating the presence of the virus, as detected using mouse anti-EHV-1 serum (Figure 3A). No such positive signals were observed in the negative control section (Figure 3B).

### 3.2. Identification of EHV-1 in the Isolates

**Figure 4:**
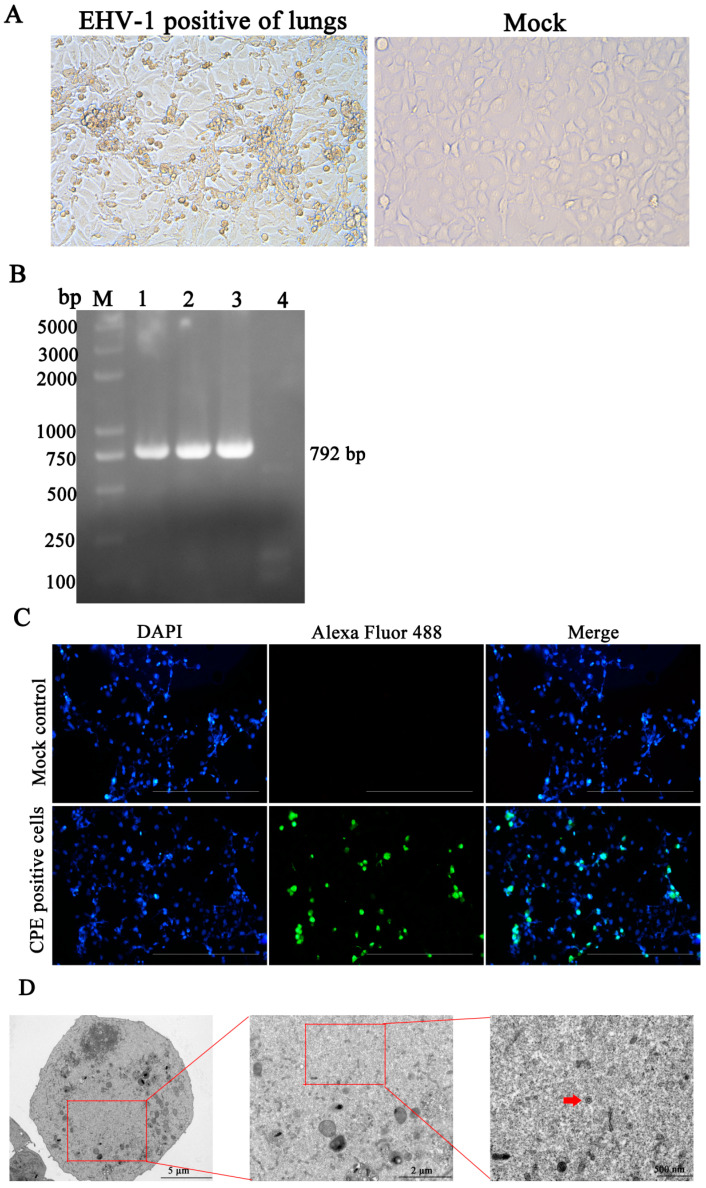
Identification of EHV-1 isolation. The RK-13 cells were inoculated with the supernatant of EHV-1-positive lung tissue (left panel) or mock control (right panel) (**A**). The cytopathogenic effect (CPE) was observed using microscopy at 48 h pi. Scale bars, 100 mm. (**B**) The gB gene of the EHV-1 isolate was confirmed by PCR. The PCR products were analyzed by 1% agarose gel. Regarding the DL2000 plus DNA marker (lane M), 1 represents EHV-1 Passage 1 cell supernatant, 2 represents EHV-1 Passage 2 cell supernatant, 3 represents EHV-1 passage 3 cell supernatant, and 4 represents negative control. (**C**) The isolate was detected by IFA. The images represent the subcellular locations of EHV-1 proteins using IFA detection with anti-EHV-1 mouse serum and the corresponding alexa fluor 488-conjugated secondary antibodies. Cells were imaged by Leica DMi8. Scale bars, 50 μm. (**D**) Transmission electron micrograph analysis. RK-13 cells were infected with LC126 (MOI = 1) and then fixed by TEM fixative at 48 hpi and observed by transmission electron microscopy. Magnified images of the regions indicated by red rectangles are demonstrated on the right. Red arrows represent EHV-1 virions.

A field strain of EHV-1 was isolated from EHV-1-positive lung tissue on susceptible cell lines. Briefly, after a 3-day incubation period, CPEs were observed in RK-13 cells (Figure 4A, left) inoculated with the EHV-1-positive lung tissue, in contrast to mock-infected cells treated with PBS (Figure 4A, right). To confirm the presence of EHV-1 isolates, supernatants from CPE-positive cells were collected, and PCR assays were performed to detect EHV-1 across different passage cycles. As shown in Figure 4B, a single band of the expected size (792 bp) was observed on a 1% agarose gel from passages P1 to P3 using specific primers for EHV-1 detection. Additionally, EHV-1 protein expression in RK-13 cells was confirmed by IFA assay using a mouse anti-EHV-1-positive serum. The isolates from the fourth passage exhibited virions both in the cytoplasm and nucleus (Figure 4C). Transmission electron microscopy (TEM) of EHV-1 virions revealed round particles approximately 110 nm in diameter, with a distinctive double-ring structure consisting of an inner nucleocapsid and an outer envelope (Figure 4D). This morphology is consistent with that of intact enveloped EHV-1 virions.

### 3.3. Phylogenetic Analysis of the ORF33 Gene

**Figure 5:**
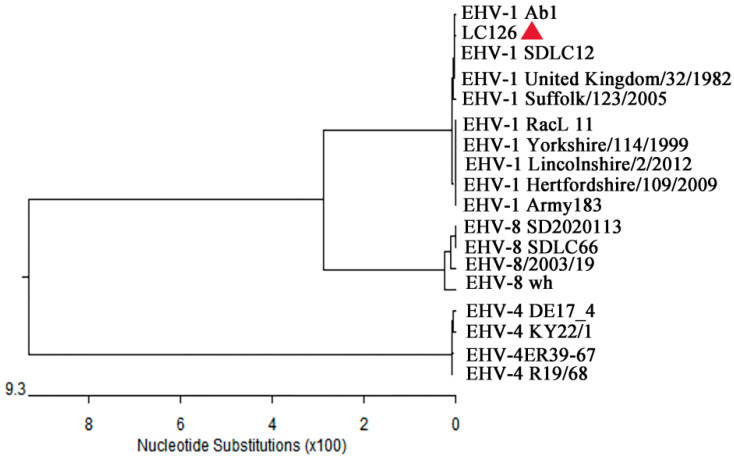
Phylogenetic tree based on the sequence of *ORF33* from the isolate in the present study and with known sequences of EHVs. Sequences of isolates of *ORF33* in the present study are labeled with “
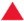
”. The scale bar indicates nucleotide substitutions per site.

The complete *ORF33* gene sequence of LC126 was obtained through PCR amplification. Phylogenetic analysis revealed that this sequence shared the closest relationship with EHV-1, particularly the EHV-1 Ab1 and SDLC12 strains, and was distinct from other known EHV-4 and EHV-8 sequences (Figure 5).

### 3.4. Replication Characteristics and Pathogenicity of EHV-1 Isolate in BALB/C Mice

**Figure 6:**
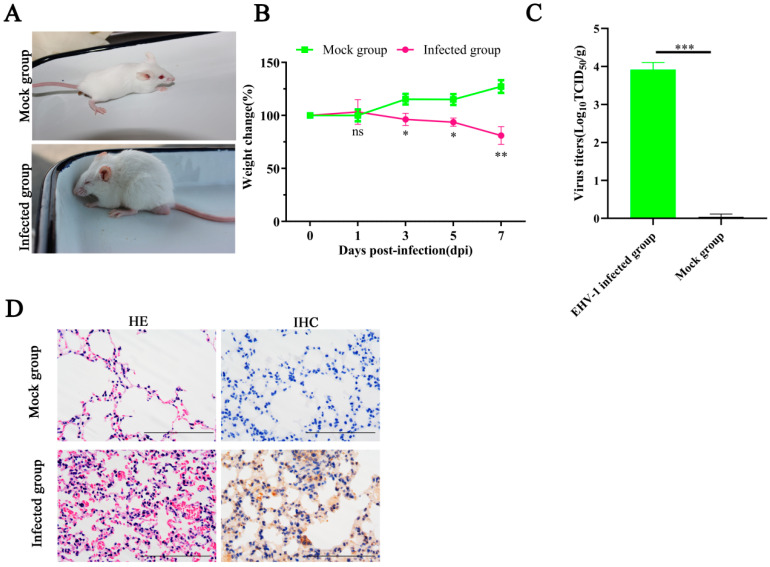
The pathogenicity of LC126 in Balb/c mice. Ten female, specific pathogen-free BALB/c mice (6 weeks old) were randomly divided into an infected group and a mock group (*n* = 5). Clinical signs (**A**), the percent change in body weight was calculated for each mouse based on the initial starting weight before virus inoculation (**B**). The lung tissues of the infected group and mock group were collected at 7 dpi to evaluate virus titers using TCID_50_. * *p* < 0.05; ** *p* < 0.01; *** *p* < 0.001. (**C**). The lung tissues of different groups of mice were collected at 7 dpi and fixed in 10% formalin solution for H&E detection (**D**, left) and IHC analysis (**D**, right).

Previous studies have demonstrated that EHV-1 can effectively replicate in the lungs and brains of BALB/c mice [16,17]. In the present study, we also assessed the replication and virulence of the LC126 strain in BALB/c mice. As shown in Figure 6, mice infected with the LC126 strain exhibited clinical signs, including depression, ruffled fur, inappetence, respiratory distress, and crouching in corners by 3 dpi, in contrast to the mock-infected group (Figure 6A). Additionally, a significant decrease in body weight was observed in the LC126-infected mice starting at 3 dpi (Figure 6B). Viral titers in the lungs of infected mice were quantified, with a mean titer of 10^3.9^TCID_50_ (Figure 6C). Histopathological analysis revealed severe lesions in the lung tissue, characterized by hyperemia and hemorrhage in LC126-infected mice (Figure 6D, left). The IHC staining was performed to detect EHV-1 antigen in the lung tissue of LC126-infected mice, using anti-EHV-1 positive serum, which confirmed the presence of viral antigen (Figure 6D, right).

## 4. Discussion

Large-scale donkey farming has seen significant growth in China in recent years, contributing to the development of the animal husbandry sector [18,19]. However, the sustainable development of the donkey industry is under threat due to the prevalence of respiratory diseases and abortion, which present considerable challenges to its continued expansion [20,21]. Notably, respiratory diseases in donkeys have been linked to various microbial agents. For instance, a study by Yang et al. [22] identified the equine influenza virus H3N8 as a significant cause of respiratory illness in donkeys. In addition, a study conducted in Shandong Province, China, reported a 21.5% positive rate (14/65) of equine influenza virus H3N8 among donkeys exhibiting respiratory diseases on large-scale farms [23]. A major concern in equine respiratory health is the role of EHV, particularly EHV-1 and EHV-4, which are well-established pathogens associated with respiratory tract infections in horses [24,25]. In addition to these viruses, several studies have implicated EHV-8 in respiratory diseases and abortion in both horses and donkeys [4,11,26]. Moreover, EAV has been recognized as a causative agent of both respiratory and reproductive diseases in equids [27,28,29]. The above-mentioned viruses were identified in the dead donkey foals. In this instance, H3N8, EAV, EHV-4, and EHV-8 were negative; only EHV-1 appears positive (Figure 2). Moreover, respiratory diseases in the donkey industry have been linked to bacterial pathogens, like *Streptococcus subsp*. *Equi* and *Rhodococcus equi*. In the present study, we first performed detection of these bacteria, but they are negative. Only EHV-1 was identified as the sole positive pathogen among the potential viral agents. While H3N8, EAV, EHV-4, and EHV-8 were all tested negative, EHV-1 was found to be present in the lung tissue of dead donkey foals (Figure 2 and Figure 3). This finding suggests that EHV-1 may be a primary pathogenic agent contributing to respiratory disease in Dezhou donkey foals (Figure 4).

Previous studies showed that EHV-1 was identified from donkeys, both with and without respiratory illness, in Ethiopia [30,31]. More recently, EHV-1 was isolated from the lung tissue of an aborted fetal donkey, further supporting its potential role in reproductive and respiratory diseases in donkeys [10]. Our study represents the first report of EHV-1 as the causative agent of respiratory disease and death in Dezhou donkey foals in Shandong Province, China. Histopathological examination of affected donkey foals revealed that EHV-1 infection led to severe alveolar swelling, inflammatory cell infiltration, and hemorrhage in the lungs (Figure 1 and Figure 3). It is noteworthy that the mice model was widely used as an animal model to detect EHVs in pathogenicity studies [3,17,32,33]. In the present study, similar pathological changes were validated and observed in a mouse model (Figure 6). Collectively, these results highlight the pathogenicity of EHV-1 in donkeys and suggest that this virus contributes significantly to respiratory disease and mortality in donkey foals. Given the impact of EHV-1 on donkey health, particularly in large-scale farming operations, it is imperative to invest in further research into effective preventative measures.

## 5. Conclusions

This study presented a case report on the EHV-1-induced infection in large-scale donkey farms in China. Our findings demonstrate that EHV-1 infection is clinically associated with respiratory distress and mortality in donkey foals. EHV-1 also causes abortion in pregnant donkeys. Given the significant impact of EHV-1 on donkey health, it is crucial for the donkey industry to implement robust biosafety management strategies to prevent the spread of this virus. Enhanced surveillance and improved biosecurity protocols are essential for mitigating the risks posed by EHV-1 and ensuring the sustainability of the donkey farming industry.

## Figures and Tables

**Table 1:** The primer sequences in this study.

Primers	Primer Sequences (5′-3′)	PCR Product Sizes
EHV-1-*gB*-F	GAACCTCAGCCAACCCA	792 bp
EHV-1-*gB*-R	GCACTTTGCGGACGAAC	
EHV-4-*gB*-F	CTTAATCGCATTTAGACCGATG	1591 bp
EHV-4-*gB* R	CCGGAACTAGAAAGATGTTATGC	
EHV-8-*gG*-F	TCAGACTGTCACTCGTGGGA	316 bp
EHV-8-*gG*-R	CCTGGAGGCCGTTTAACACA	
EAV-*N*-F	ATGGCGTCAAGACGATCAC	333 bp
EAV-*N*-R	TTACGGCCCTGCTGGAGGC	
H3N8-*HA*-F	ATTCATCACCCGAGTTCAAA	595 bp
H3N8-*HA*-R	TTTCATTAATCTGGTCGATGGC	
H3N8-*M*-F	ACTGTGACAACCGAAGTG	224 bp
H3N8-*M*-R	TGCCTGGCCTTACTAGC	
EHV-1-*ORF33*-F	ATGTCCTCTGGTTGCCGTTC	2943 bp
EHV-1-*ORF33*-R	TTAAACCATTTTTTCATTTTC	

## Data Availability

The data supporting this study’s findings are available from the corresponding author upon reasonable request.

## References

[1] Afify A.F., Hassanien R.T., El Naggar R.F., Rohaim M.A., Munir M. (2024). Unmasking the ongoing challenge of equid herpesvirus-1 (EHV-1): A comprehensive review. Microb. Pathog..

[2] Tau R.L., Ferreccio C., Bachir N., Torales F., Romera S.A., Maidana S.S. (2023). Comprehensive analysis of equid herpesvirus recombination: An insight into the repeat regions. J. Equine Vet. Sci..

[3] Wang T., Hu L., Liu M., Wang T., Hu X., Li Y., Liu W., Li Y., Wang Y., Ren H. (2022). The emergence of viral encephalitis in donkeys by equid herpesvirus 8 in China. Front. Microbiol..

[4] Garvey M., Suarez N.M., Kerr K., Hector R., Moloney-Quinn L., Arkins S., Davison A.J., Cullinane A. (2018). Equid herpesvirus 8: Complete genome sequence and association with abortion in mares. PLoS ONE.

[5] Doll E.R., Wallace E., Richards M.G. (1954). Thermal, hematological, and serological responses of weanling horses following inoculation with equine abortion virus: Its similarity to equine influenza. Cornell Vet..

[6] Oladunni F.S., Horohov D.W., Chambers T.M. (2019). EHV-1: A constant threat to the horse industry. Front. Microbiol..

[7] Chen L., Li S., Li W., Yu Y., Sun Q., Chen W., Zhou H., Wang C., Li L., Xu M. (2024). Rutin prevents EqHV-8-induced infection and oxidative stress via Nrf2/HO-1 signaling pathway. Front. Cell. Infect. Microbiol..

[8] Li L., Cui X., Yu Y., Sun Q., Li W., Li Y., Li S., Chen L., Khan M.Z., Wang C. (2024). Blebbistatin as a novel antiviral agent targeting equid herpesvirus type 8. Front. Vet. Sci..

[9] Li S., Li L., Sun Y., Khan M.Z., Yu Y., Ruan L., Chen L., Zhao J., Jia J., Li Y. (2024). Protective role of cepharanthine against equid herpesvirus type 8 through AMPK and Nrf2/HO-1 pathway activation. Viruses.

[10] Tong P., Pan J., Dang Y., Yang E., Jia C., Duan R., Tian S., Palidan N., Kuang L., Wang C. (2024). First identification and isolation of equine herpesvirus type 1 in aborted fetal lung tissues of donkeys. Virol. J..

[11] Wang T., Xi C., Yu Y., Liu W., Akhtar M.F., Li Y., Wang C., Li L. (2023). Characteristics and epidemiological investigation of equid herpesvirus 8 in donkeys in Shandong, China. Arch. Virol..

[12] Wang T., Hu L., Wang Y., Liu W., Liu G., Zhu M., Zhang W., Wang C., Ren H., Li L. (2022). Identification of equine herpesvirus 8 in donkey abortion: A case report. Virol. J..

[13] Wang T., Du Q., Niu Y., Zhang X., Wang Z., Wu X., Yang X., Zhao X., Liu S.L., Tong D. (2019). Cellular p32 is a critical regulator of porcine circovirus type 2 nuclear egress. J. Virol..

[14] Li L., Wang J., Chen L., Ren Q., Akhtar M.F., Liu W., Wang C., Cao S., Liu W., Zhao Q. (2024). Diltiazem HCl suppresses porcine reproductive and respiratory syndrome virus infection in susceptible cells and in swine. Vet. Microbiol..

[15] Wang T., Hu L., Li R., Ren H., Li S., Sun Q., Ding X., Li Y., Wang C., Li L. (2024). Hyperoside inhibits EHV-8 infection via alleviating oxidative stress and IFN production through activating JNK/Keap1/Nrf2/HO-1 signaling pathways. J. Virol..

[16] Fuentealba N.A., Sguazza G.H., Zanuzzi C.N., Bravi M.E., Scrochi M.R., Valera A.R., Corva S.G., Gimeno E.J., Pecoraro M.R., Galosi C.M. (2019). Immunoprotective response induced by recombinant glycoprotein D in the BALB/c respiratory mouse model of Equid alphaherpesvirus 1 infection. Rev. Argent. Microbiol..

[17] Walker C., Ruitenberg K.M., Love D.N., Millar Whalley J. (2000). Immunization of BALB/c mice with DNA encoding equine herpesvirus 1 (EHV-1) glycoprotein D affords partial protection in a model of EHV-1-induced abortion. Vet. Microbiol..

[18] Zhang Z., Huang B., Wang Y., Zhu M., Liu G., Wang C. (2023). A survey report on the donkey original breeding farms in China: Current aspects and future prospective. Front. Vet. Sci..

[19] Seyiti S., Kelimu A. (2021). Donkey industry in China: Current aspects, suggestions and future challenges. J. Equine Vet. Sci..

[20] Li L., Li S., Ma H., Akhtar M.F., Tan Y., Wang T., Liu W., Khan A., Khan M.Z., Wang C. (2024). An overview of infectious and non-infectious causes of pregnancy losses in equine. Animals.

[21] Rickards K.J., Thiemann A.K. (2019). Respiratory disorders of the donkey. Vet. Clin. North Am. Equine Pract..

[22] Yang H., Xiao Y., Meng F., Sun F., Chen M., Cheng Z., Chen Y., Liu S., Chen H. (2018). Emergence of H3N8 equine influenza virus in donkeys in China in 2017. Vet. Microbiol..

[23] Yongfeng Y., Xiaobo S., Nan X., Jingwen Z., Wenqiang L. (2020). Detection of the epidemic of the H3N8 subtype of the equine influenza virus in large-scale donkey farms. Int. J. Vet. Sci. Med..

[24] Pusterla N., Sandler-Burtness E., Barnum S., Hill L.A., Mendonsa E., Khan R., Portener D., Ridland H., Schumacher S. (2022). Frequency of detection of respiratory pathogens in nasal secretions from healthy sport horses attending a spring show in California. J. Equine Vet. Sci..

[25] Pusterla N., Leutenegger C.M., Barnum S., Wademan C., Hodzic E. (2021). Challenges in navigating molecular diagnostics for common equine respiratory viruses. Vet. J..

[26] Liu C., Guo W., Lu G., Xiang W., Wang X. (2012). Complete genomic sequence of an equine herpesvirus type 8 Wh strain isolated from China. J. Virol..

[27] Otzdorff C., Beckmann J., Goehring L.S. (2021). Equine arteritis virus (EAV) outbreak in a show stallion population. Viruses.

[28] Rivas J., Neira V., Mena J., Brito B., Garcia A., Gutierrez C., Sandoval D., Ortega R. (2017). Identification of a divergent genotype of equine arteritis virus from South American donkeys. Transbound. Emerg. Dis..

[29] Stadejek T., Mittelholzer C., Oleksiewicz M.B., Paweska J., Belak S. (2006). Highly diverse type of equine arteritis virus (EAV) from the semen of a South African donkey: Short communication. Acta Vet. Hung..

[30] Negussie H., Gizaw D., Tesfaw L., Li Y., Oguma K., Sentsui H., Tessema T.S., Nauwynck H.J. (2017). Detection of equine herpesvirus (EHV)-1, -2, -4, and -5 in Ethiopian equids with and without respiratory problems and genetic characterization of EHV-2 and EHV-5 strains. Transbound. Emerg. Dis..

[31] Temesgen T., Getachew Y., Negussie H. (2021). Molecular identification of equine herpesvirus 1, 2, and 5 in equids with signs of respiratory disease in central Ethiopia. Vet. Med..

[32] Mesquita L.P., Arévalo A.F., Zanatto D.A., Miyashiro S.I., Cunha E.M.S., de Souza M.D.C.C., Villalobos E.M.C., Mori C.M.C., Maiorka P.C., Mori E. (2017). Equine herpesvirus type 1 induces both neurological and respiratory disease in Syrian hamsters. Vet. Microbiol..

[33] Abas O., Abdo W., Kasem S., Alwazzan A., Saleh A.G., Saleh I.G., Fukushi H., Yanai T., Haridy M. (2020). Time Course-Dependent Study on Equine Herpes Virus 9-Induced Abortion in Syrian Hamsters. Animals.

